# Composite scattering characteristics analysis of micro-ellipsoidal periodic structure optical surface and microdefects

**DOI:** 10.1016/j.heliyon.2024.e36039

**Published:** 2024-08-08

**Authors:** Zhi-qiang Yang, Juan Chen, Li-guo Wang, Li-hong Yang, Yao Li, Zhen-sen Wu, Lei Gong

**Affiliations:** a School of Photoelectrical Engineering, Xi'an Technological University, shaanxi 710021, China; b School of Physics and Optoelectronic Engineering, Xidian University, Xi'an, shaanxi 710016, China

**Keywords:** Light scattering, Micro-ellipsoidal periodic structure optical surface, Microdefects, Field distribution

## Abstract

In order to adjust and detect micro-nano periodic structure optical surface accurately and efficiently, the problem of composite scattering between micro-ellipsoidal periodic structure optical surface and pore defects is studied use the multi-resolution time domain (MRTD) approach. A calculation model is established for the intensity distribution of composite scattering, which is modulated by the micro-ellipsoidal periodic structure optical surface and microdefects. Results are in good agreement with those obtained using CST Microwave Studio software and the finite-different time-domain (FDTD) approach, which demonstrates the effectiveness of the calculation model and method. By combining the field distribution of the micro-ellipsoidal periodic structure optical surface containing microdefects with the optical response at different wavelengths, it is necessary to study the influence of various parameters of the micro-ellipsoidal structure and microdefects on the optical system of metamaterials. The effects of the parameters such as roughness, structure of micro-ellipsoidal unit, defect sizes and buried depths on the composite scattering characteristics are analyzed numerically. The results provide technical support for the fields of functional surface design, ultrasensitive detection, scattering peak orientation and frequency selection.

## 1 Introduction

Micro- and nano-optical periodic metasurfaces have significant academic importance and wide applications in various fields, including the design of functional metasurfaces and modulation of optical fields. The rational design of the superstructure morphology and its spatial arrangement can enable the periodic structure optical surfaces to achieve different functions, such as superlens imaging [[1], [2], [3], [4], [5]], holographic imaging [[6], [7], [8], [9]] and color filtering [10,11]. Purity and quality requirements of periodic structure optical surfaces have been rapidly improved with the rapid development of optical metasurface (see Table 1).

**Table 1:** Connection coefficients of different orders of the Daubechies scale function.

ν	$D_{1}$	$D_{2}$	$D_{3}$	$D_{4}$
0	1	1.2291666667	1.2918129281	1.3110340773
1		−0.0937500000	−0.1371343456	−0.1560100710
2		0.0104166667	0.0287617723	0.0419957460
3			−0.0034701413	−0.0086543236
4			0.0000080265	0.0008308695
5				0.0000108999
6				−0.0000000041
First-order moment $M_{1}$		0.6339743121		1.0053923835

The interaction between micro nano periodic super structure and light has important academic significance and wide applications in various functional metamaterials design, optical field manipulation and target optical characteristic control [[12], [13], [14], [15]]. In high-energy metamaterial laser systems, as the scale of the periodic structure gradually reaches the nanometer scale, the inadvertent introduction of micro-nano-scale defects such as bubbles, scratches, etc., will interact with the micro- and nano-periodic structural units during processing, lossing the transmitted light energy and producing distortions in the wavefront, which will bring negative effects such as scattering angular spectral broadening, affecting the performance of the entire optical system seriously [16,17].The detection of defect particles is one of the most important research focuses in the aspects of the preparation, test and performance evaluation of the periodic structure optical surface. However, there are few studies on this aspect currently.

In recent years, extensive research work has been conducted based on the unique optical properties of superstructures using different methods. Ray [18] et al. plated a de-wetted metal film to form a dry etching mask and confirmed the precise control of light propagation on metasurface. Abdallah et al. [19,20] prepared TiN, TiAlVN and Ti6Al4V films by DC magnetron sputtering technology, and the prepared films can prevent the erosion of corrosive media. Rihawy [21] et al. used arc discharge deposition method to deposit aluminum nitride films on silicon substrate, which plays an important role in characterizing the thickness and chemical composition of the films. Gallant et al. [22] controlled the growth of crystal geometry and developed a superstructure surface with nanoscale anisotropic spiral structure. Tiejun et al. [[23], [24], [25]] has proposed the concept of random metasurface and demonstrated various control effects, such as random scattering and polarization conversion in different wavebands with diffuse scattering effects on incident electromagnetic waves in all directions. Wei and Zhixiang et al. [26] proposed various new structures, such as metal-insulator-metal (MIM) waveguides coupled in parallel with plasma at the metal-insulator (MI) interface. However, defects inadvertently introduced during the actual manufacturing process, which are comparable to the order of magnitude of periodic units and can seriously affect the lens imaging effect, receive less consideration in the actual research process.

Previous studies on metamaterials and metasurfaces have mainly focused on performance realization, design, and manufacturing. They have paid little attention to the physical mechanism and properties of metasurfaces and deep layers of materials. Additionally, they have not taken into account the defects inadvertently introduced during the actual manufacturing process, which are equivalent in magnitude to periodic units. One of the focuses of this paper is to discuss and analyze the influence of the defect physical parameters on the metasurfaces coupling light field.

Due to the precise manipulation of materials' microstructure at the nanoscale, the process of fabricating metasurfaces is often time-consuming and expensive. As we all know, light scattering is an effective non-contact method for assessing optical surface quality, which can reduce experimental and manufacturing costs. To enhance the performance of metasurfaces and reduce the cost at the same time, numerous scholars have started their work from theoretical studies, such as plane-wave expansion method (PWE) [27], finite element method (FEM) [28], rigorous coupled wave analysis (RCWA) [29], finite difference time domain (FDTD) [30], etc.

In this paper, we will utilize the multi-resolution time domain (MRTD) approach, which not only has good dispersion characteristics, but also selects and expands the scale function and wavelet function based on the rate of change of field values, thus greatly saving computer memory by reducing the sampling rate of spatial grid, and solving the contradiction between computational volume and computational accuracy in the FDTD method. Therefore, it is a powerful technique to calculate scattering fields around the complex nanostructure particles.

This article is organized as follows. In Section 2, the schematic diagram of composite scattering model between micro-ellipsoidal periodic structure optical surface and microdefects are shown. The composite scattering problem between periodic structure optical surface and defects is studied based on the MRTD approach. In Section 3, we present simulation results on the composite scattering characteristics of microdefects and optical surfaces with different structures of Micro-Ellipsoidal units, defect sizes, and buried depths. Concluding remarks are made in the last section.

## 2 Scattering model and MRTD method key technology

### 2.1 Micro-ellipsoidal periodic structure optical surface and microdefects composite scattering model

The defects in a series of processes such as cleaning, gluing, smoothing, exposing, and etching are the main sources of failure in the performance of micro/nano periodic optical systems. In this paper, the composite scattering characteristics of periodic structure optical surface and pore defects are the main research contents.

**Fig. 1:**
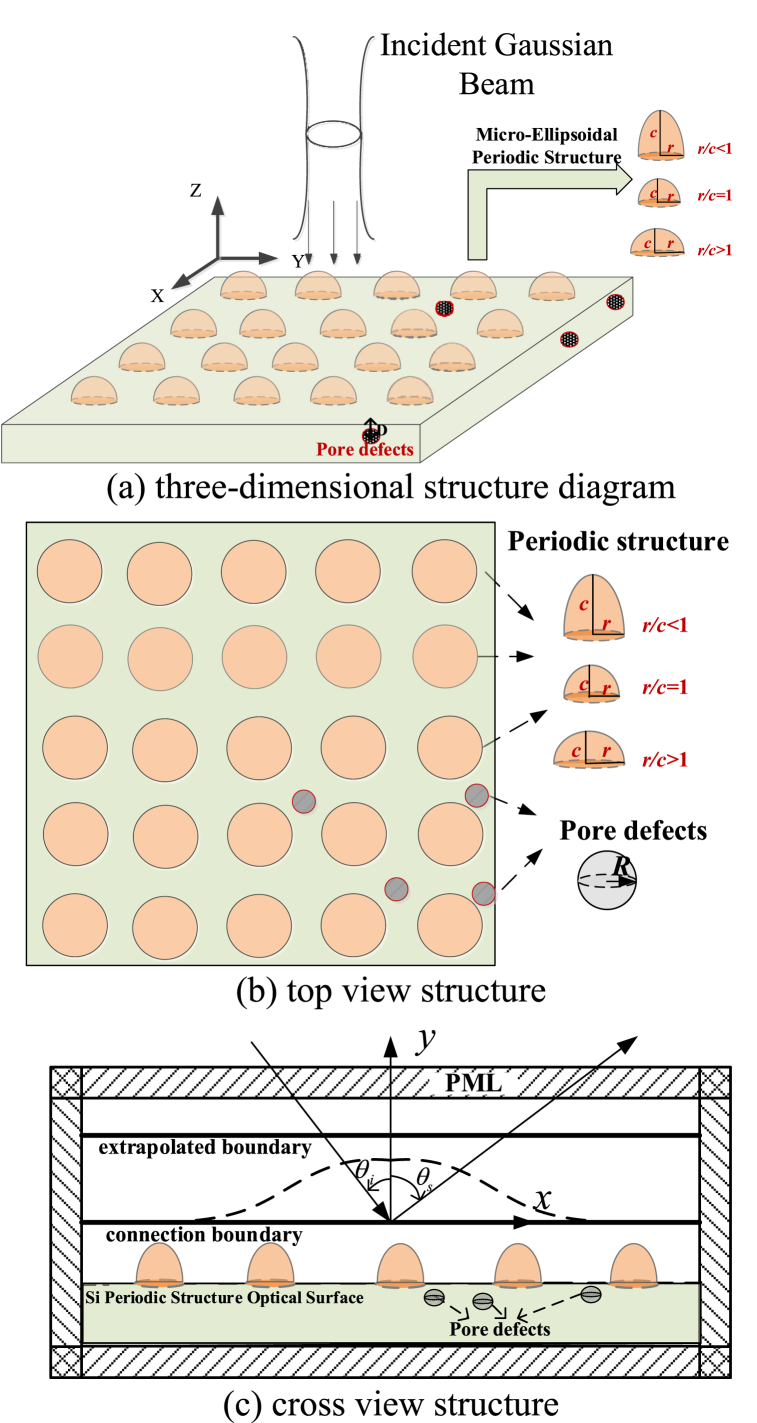
Schematic diagram of coupled scattering model between metasurface and microdefects.

The schematic diagram of the composite scattering model between metasurface and microdefects is shown in Fig. 1. Fig. 1(a) depicts the three-dimensional structure, while Fig. 1(b) and (c) illustrate the structure in the top and cross view. In this paper, the micro-nano surface of an ellipsoidal unit doped with oxide defects inadvertently generated during the manufacturing process is selected as the research object.

In considering the flexibility of the hemispherical structure of the superstructure surface, the unit structure can be expanded into a polymorphic rotating ellipsoid by changing the ratio of the long and short axes, as shown in Fig. 1 *c/r* > 1 is characterized as an oblate rotating ellipsoidal periodic structure. *c/r* < 1 is characterized as a flat rotating ellipsoidal periodic structure, and *c/r* = 1 is characterized as a spherical particle periodic structure. It can be further extended to other typical metasurfaces for various engineering applications.

In this paper, defect radius is R and burial depth is D. The wavelength of the incident wave is λ and wave number k = 2π/λ, and the power of the incident Gaussian beam is P = 200 mW. The incident angle of the incident wave is θi and scattering angle is *θs*. The basic theoretical framework of this paper is based on C++.

### 2.2 Deduction of coupled scattering field based on MRTD

By combining the constitutive relations of the medium with Maxwell's curl equations, we can derive the differential forms for each component of the electric and magnetic fields as follows:

(1) $$\begin{array}{l} \frac{\partial H_{z}}{\partial y}-\frac{\partial H_{y}}{\partial z}=\varepsilon \frac{\partial E_{x}}{\partial t}+\sigma E_{x}\\ \frac{\partial H_{x}}{\partial z}-\frac{\partial H_{z}}{\partial x}=\varepsilon \frac{\partial E_{y}}{\partial t}+\sigma E_{y}\\ \frac{\partial H_{y}}{\partial x}-\frac{\partial H_{x}}{\partial y}=\varepsilon \frac{\partial E_{z}}{\partial t}+\sigma E_{z} \end{array}\}$$

(2) $$\begin{array}{l} \frac{\partial E_{z}}{\partial y}-\frac{\partial E_{y}}{\partial z}=-\mu \frac{\partial H_{x}}{\partial t}-\sigma_{m}H_{x}\\ \frac{\partial E_{x}}{\partial z}-\frac{\partial E_{z}}{\partial x}=-\mu \frac{\partial H_{y}}{\partial t}-\sigma_{m}H_{y}\\ \frac{\partial E_{y}}{\partial x}-\frac{\partial E_{x}}{\partial y}=-\mu \frac{\partial H_{z}}{\partial t}-\sigma_{m}H_{z} \end{array}\}$$

Based on the expansion of Maxwell's curl equation, taking the electric field in *x* direction as an example, the electromagnetic field components are expanded by the Daubechies scale function and the rectangular pulse function in time domain, obtaining the following formula:

(3) $$E_{x}\left(\vec{r},t\right)=\sum_{i,j,k,n=-\infty }^{\infty }E_{i+0.5,j,k}^{\phi x,n}\phi_{i+0.5}\left(x\right)\phi_{j}\left(y\right)\phi_{k}\left(z\right)h_{n}\left(t\right)$$

(4) $$H_{y}\left(\vec{r},t\right)=\sum_{i,j,k,n=-\infty }^{\infty }H_{i+0.5,j,k+0.5}^{\phi y,n+0.5}\phi_{i+0.5}\left(x\right)\phi_{j}\left(y\right)\phi_{k+0.5}\left(z\right)h_{n+0.5}\left(t\right)，$$

(5) $$H_{z}\left(\vec{r},t\right)=\sum_{i,j,k,n=-\infty }^{\infty }H_{i+0.5,j+0.5,k}^{\phi z,n+0.5}\phi_{i+0.5}\left(x\right)\phi_{j+0.5}\left(y\right)\phi_{k}\left(z\right)h_{n+0.5}\left(t\right)，$$

Where, $E_{i+0.5,j,k}^{\phi x,n}$, $H_{i+0.5,j,k+0.5}^{\phi y,n+0.5}$ and $H_{i+0.5,j+0.5,k}^{\phi z,n+0.5}$ are field expansion coefficients. *i*, *j*, *k* and *n* represent three-dimensional spatial coordinates and time scales. The corresponding discrete formulas are t=nΔt, x=iΔx, y=jΔy, z=kΔz, where Δx, Δy, Δz and Δt represent discrete intervals between space and time respectively.

For the convenience of Garlerkin test, $h_{n}\left(t\right)$ and $\phi_{m}\left(\nu \right)$ is defined as follows:

(6) $$h_{n}\left(t\right)=h\left(t/\Delta t-n\right)，$$

(7) $$\phi_{i}\left(v\right)=\phi \left(v/\Delta v-i\right),v=x,y,z，$$

Where, $h_{n}\left(t\right)$ and $\phi_{i}\left(\nu \right)$ respectively represent the rectangular pulse function and the Daubechies wavelet function with tight support domain. For the N order Daubechies wavelet function $D_{N}$, the tight support domain is [0, 2N-1], indicating that the connection coefficient a(ν) has values only in a finite region, and the values in external region are all 0, and the support region is [-(2N-1), (2N-1)-1], using Ls to represent 2N-1 and regarding it as the effective support size of the wavelet basis function.

When N = 1, the Daubechies scale function degenerates to the Haar scale function, assuming that when N = 2, for the Daubechies-2 wavelet function, its tight support domain is [0,3], i.e, Ls = 3. When ν>2, it can be known from the concept of tight support domain that a(ν)=0; and when l<0, a(ν) has a symmetry relationship that a(−1−ν)=a(−ν), so only the values of a(l) in the interval 0≤l≤2 need to be obtained.

$\phi_{i+0.5}\left(x\right)\phi_{j}\left(y\right)\phi_{k}\left(z\right)h_{n+0.5}\left(t\right)$ are selected as test functions to perform standard Galerkin test. Performing an inner product operation with the field yields, which allows for obtaining:

$$\sum_{i^{\prime },j^{\prime },k^{\prime },n^{\prime }=-\infty }^{\infty }\varepsilon E_{i^{\prime }+0.5,j^{\prime },k^{\prime }}^{\phi x,n^{\prime }}<\phi_{i^{\prime }+0.5}\left(x\right),\phi_{i+0.5}\left(x\right)><\phi_{j^{\prime }}\left(y\right),\phi_{j}\left(y\right)><\phi_{k^{\prime }}\left(z\right),\phi_{k}\left(z\right)><\frac{\partial h_{n^{\prime }}\left(t\right)}{\partial t},h_{n+0.5}\left(t\right)>+\sum_{i^{\prime },j^{\prime },k^{\prime },n^{\prime }=-\infty }^{\infty }\sigma E_{i^{\prime }+0.5,j^{\prime },k^{\prime }}^{\phi x,n^{\prime }}<\phi_{i^{\prime }+0.5}\left(x\right),\phi_{i+0.5}\left(x\right)><\phi_{j^{\prime }}\left(y\right),\phi_{j}\left(y\right)><\phi_{k^{\prime }}\left(z\right),\phi_{k}\left(z\right)><h_{n^{\prime }}\left(t\right),h_{n+0.5}\left(t\right)>$$

(8) $$\begin{array}{l} \;=\sum_{i^{\prime },j^{\prime },k^{\prime },n^{\prime }=-\infty }^{\infty }H_{i^{\prime }+0.5,j^{\prime }+0.5,k^{\prime }}^{\phi z,n^{\prime }+0.5}<\phi_{i^{\prime }+0.5}\left(x\right),\phi_{i+0.5}\left(x\right)><\frac{\partial \phi_{j^{\prime }+0.5}\left(y\right)}{\partial y},\phi_{j}\left(y\right)><\phi_{k^{\prime }}\left(z\right),\phi_{k}\left(z\right)><h_{n^{\prime }+0.5}\left(t\right),h_{n+0.5}\left(t\right)>\\ -\sum_{i^{\prime },j^{\prime },k^{\prime },n^{\prime }=-\infty }^{\infty }H_{i^{\prime }+0.5,j^{\prime },k^{\prime }+0.5}^{\phi y,n^{\prime }+0.5}<\phi_{i^{\prime }+0.5}\left(x\right),\phi_{i+0.5}\left(x\right)><\phi_{j^{\prime }}\left(y\right),\phi_{j}\left(y\right)><\frac{\partial \phi_{k^{\prime }+0.5}\left(z\right)}{\partial z},\phi_{k}\left(z\right)><h_{n^{\prime }+0.5}\left(t\right),h_{n+0.5}\left(t\right)> \end{array}$$

After calculation and simplification, the expansion coefficient of the electric field component in the x direction of the program can be obtained:

(9) $$E_{i+0.5,j,k}^{\phi_{x,n+1}}=\frac{2\varepsilon -\sigma \Delta t}{2\varepsilon +\sigma \Delta t}E_{i+0.5,j,k}^{\phi x,n}+\frac{2}{2\varepsilon +\sigma \Delta t}\sum_{v=-L_{s}}^{L_{s}-1}a\left(v\right)\cdot \left(H_{i+0.5,j+v+0.5,k}^{\phi z,n+0.5}\frac{\Delta t}{\Delta y}-H_{i+0.5,j,k+v+0.5}^{\phi y,n+0.5}\frac{\Delta t}{\Delta z}\right)$$

Finally, the fields are corrected in all directions. Taking *E*_*x*_ as an example, the Ex correction iteration formula is as follows (other regions can refer to this direction for correction):

Ei+0.5,j,kϕx,n+1scat=Ei+0.5,j,kϕx,nscat+Δt/εΔy∑v=−LsLs−1a(v)Hi+0.5,j+v+0.5,kϕz,n+0.5scat

(10) −Δt/εΔz[∑v=−LsKmin−k−1a(v)Hi+0.5,j,k+v+0.5ϕy,n+0.5scat+∑v=Ktmin−kLs−1a(v)(Hi+0.5,j,k+v+0.5ϕy,n+0.5tot−Hi+0.5,j,k+v+0.5ϕy,n+0.5inc)

Where, $i\in \left[I_{tmin},I_{t\;\max}-1\right],j\in \left[J_{tmin},J_{tmax}\right],k\in \left[K_{t\;\min}-L_{s}+1,K_{t\;\min}-1\right]$.The superscript “scat”, “tot”and “inc”represent the scattering field, the total field, and the incident field respectively.

In MRTD calculations, similar to FDTD, achieving numerical stability is necessary, which requires the time step Δt to satisfy the numerical stability condition in equation (11):

(11) $$v_{\max}\Delta t\leq {\sum_{l=-L_{s}}^{L_{s}-1}\left|a\left(l\right)\right|}^{-1}{\left(\frac{1}{\Delta x^{2}}+\frac{1}{\Delta y^{2}}+\frac{1}{\Delta z^{2}}\right)}^{-\frac{1}{2}}$$

In the equation, $v_{\max}$ represents the maximum velocity of electromagnetic wave propagation within a computational domain. The multi-resolution time-domain finite difference algorithm, derived from Maxwell's equations, employs spatial and temporal differences instead of differentiation, which inevitably introduces errors. To ensure precision, the spatial step sizes Δx, Δy, Δz and should be sufficiently small; typically chosen as 1/20 to 1/10 of the wavelength corresponding to the highest calculated frequency. Moreover, the selection of a time step size must satisfy numerical stability conditions in order to prevent divergence of field quantities with increasing time steps.

## 3 Numerical simulation and discussion

### 3.1 Validation of theory and codes

**Fig. 2:**
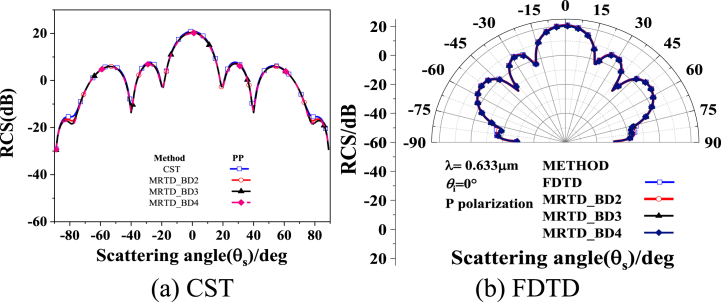
Comparison of MRTD and FDTD, CST results.

To validate the theory, the results are compared with numerical results obtained using Computer Simulation Technology (CST) Microwave Studio and FDTD method. CST Microwave Studio is one of the most effective and accurate professional simulation packages for engineers designing 3D electromagnetic, microwave circuits and temperature fields. Furthermore, our laboratory is the Sino-Germany Joint CST training center in northwestern China. Fig. 2 displays the calculated results of the Three-dimensional Micro-Ellipsoidal Periodic Structure Optical Surface with a 5*5 array arrangement, where the wavelength λ is 0.633 μm, the incident angle is $\theta_{i}=0^{\circ }$, P-polarization, and the rotation radius and long axis of the Micro-Ellipsoidal unit are 2.5λ μm and 4.0λ μm, respectively. The distance between the centers of adjacent unit is L = 8.2λ μm. The CST, FDTD and MRTD calculations are performed and the results are compared as follow.

As can be seen from Fig. 2(a) and (b), the results based on the MRTD method, where the MRTD method partly incorporates three different order vanishing moment Daubechies wavelet functions, are in good agreement with the CST and FDTD results. The previous work of our group has shown that P-polarized light is recommended in the metasurfaces quality and characteristics detection, while P-polarized light is more advantageous to obtain defect scattering field and analyze its characteristics [31], so the numerical algorithm in the paper only discusses the affection of the P polarized scattering field.

### 3.2 Field distribution and spectral response of the micro-ellipsoidal metasurfaces containing microdefects

**Fig. 3:**
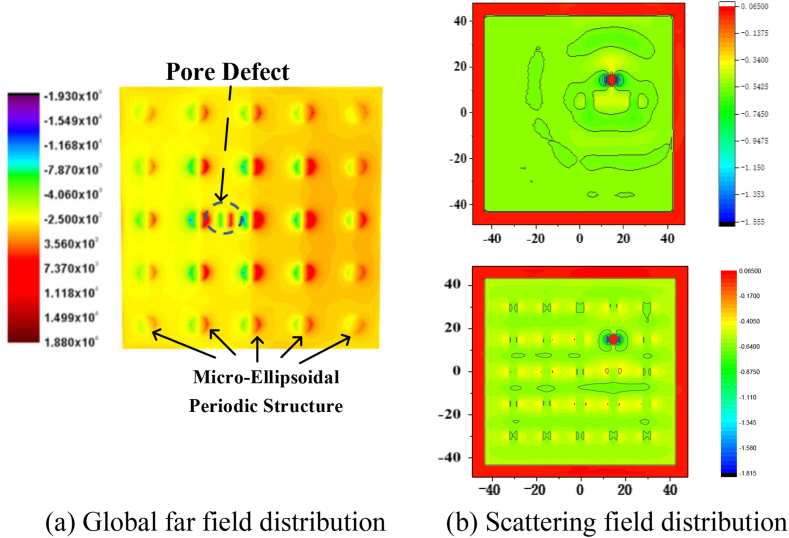
Field distribution of single crystal silicon ellipsoid periodic structure optical surface containing defect particles.

Based on section 3.1, Fig. 3(a) and (b) adds the vacuum pore defect of size R = 1.0λ μm, and the other parameters are the same as Fig. 3. As shown in Fig. 3(b), the color of the field values changes sharply to red (see black circles in Fig. 3(a)) and the field strength increases significantly where the defects appear. From the physical representation, defects break the symmetry in the micro and nano superstructure and greatly affects the field distribution on the superstructure surface, which in turn has a significant impact on the overall performance of the optical system. Thus, the presence of defects cannot be ignored. On the contrary, in many fields, the presence of defects can be used to control the optical field precisely, achieve localized light field enhancement, and other meaningful work. Therefore, in order to analyze the influence and contribution of defects to the optical field control of metasurfaces more precisely, we will use the MRTD method to carry out three-dimensional micro-ellipsoidal metasurfaces and pore defects calculations from Section 3.3. We will analyze the influence of the structure of the rotating ellipsoidal periodic unit and the physical parameters of defects on the distribution of optical fields.

**Fig. 4:**
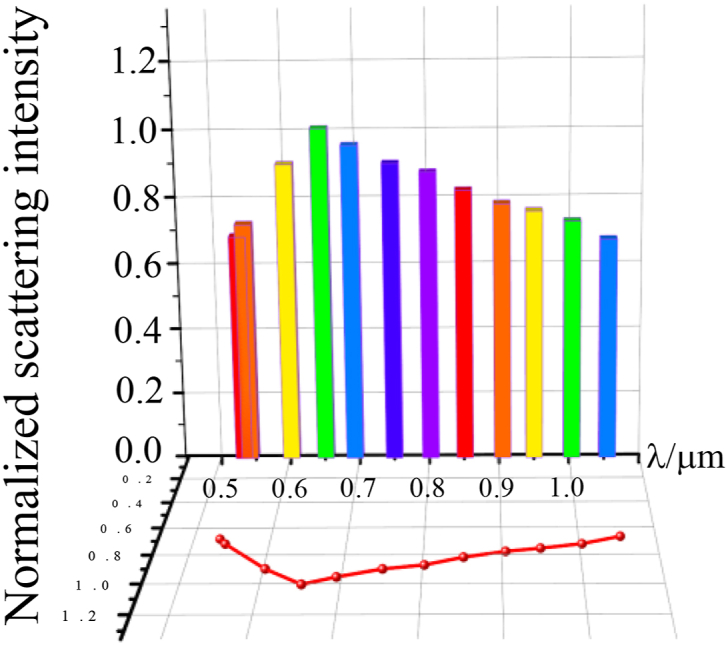
Optical response of optical surface with different wavelength.

The response of the optical metasurfaces varies significantly across different wavelengths. The relationship between the optical response with metasurfaces and the wavelength is shown in Fig. 4. Results show that the intensity of electromagnetic scattering and optical response of the optical surface increases significantly when the λ = 0.633 μm. So the wavelength of simulation works will be chosen as 0.633 μm.

### 3.3 The influence of roughness parameters on the scattering field of superstructure surface

**Fig. 5:**
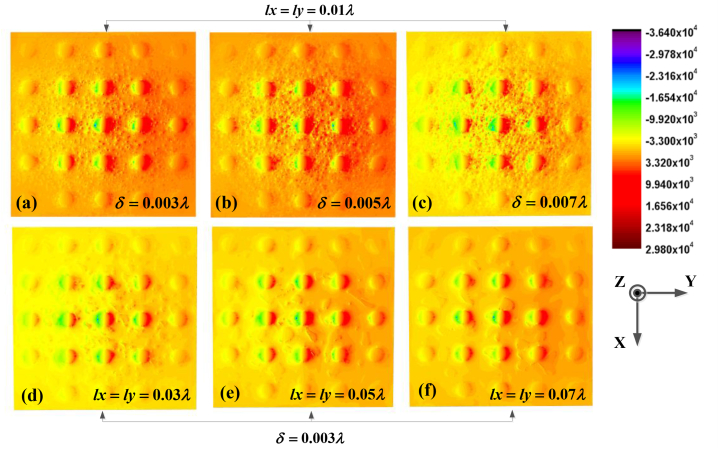
The influence of roughness on the field distribution on the metasurface.

Fig. 5 (a)(b)(c) illustrate how changing the root mean square height affects the intensity distribution of the light field, while keeping the correlation length fixed and unchanged. The Figures illustrates that an increase in the root mean square height leads to a strong disturbance on the optical surface. The interaction between incident waves and rough surfaces present on this interface induces variations on the metasurface. This change is determined by the amplitude of the rough surface, which is directly proportional to the root mean square height. In other words, an increase in root mean square height leads to oscillation and instability of the light field, resulting in a greater impact on the surface light field and a decrease in maximum field value of metasurface. The stronger the interaction between each rough point in practice, the larger the root mean square height. In the manufacturing process of metasurface, the presence of roughness on the surface are depended on the manufacturing process.

Fig. 5 (d)(e)(f) depict the impact of varying correlation length while maintaining a constant root mean square height. According to the definition of roughness, the correlation length represents the distance over which two rough points on a surface exhibit correlation. Given the characteristics of a rough surface, it can be observed from the graph that there is also an increase in the distribution of light scattering intensity on metasurface with an increasing correlation length between points on the surface. This is determined by the definition of the correlation length in rough surface theory, which can be elucidated as follows: a larger correlation length implies a greater distance between each rough point on the metasurface, resulting in diminished interaction and reduced impact on the surface optical field. From physical representation, the scattering contribution of interface roughness in this case tends to independently contribute to the overall field. Therefore, it can be concluded that the correlation length and root mean square height of a rough surface are crucial parameters which determine the overall performance.

### 3.4 The influence of shapes of ellipsoid *c/r* on the scattering field of superstructure surface

**Fig. 6:**
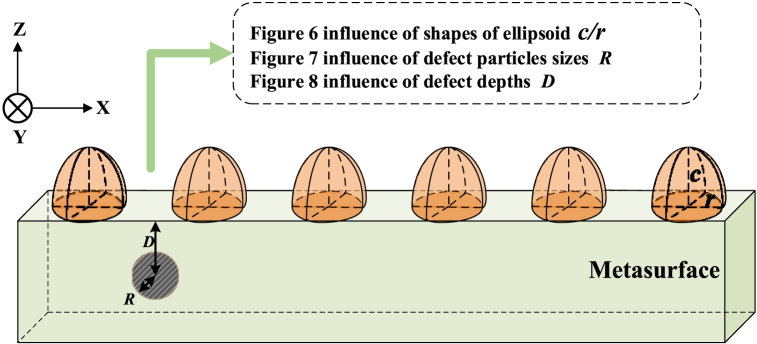
Illustration of superstructure surface unit.

**Fig. 7:**
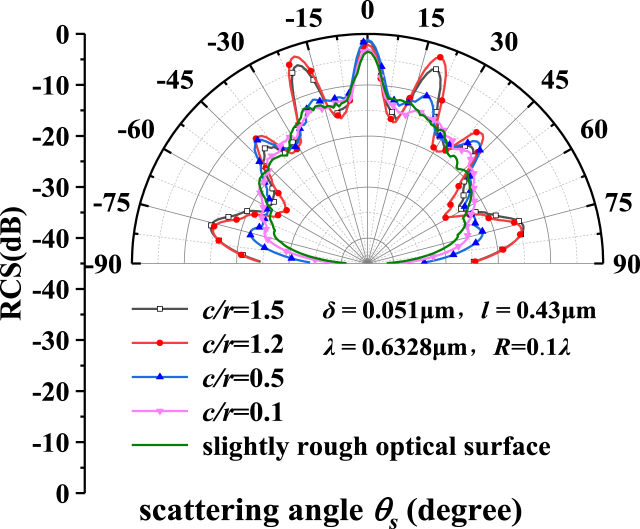
Bistatic radar cross.

**Fig. 8:**
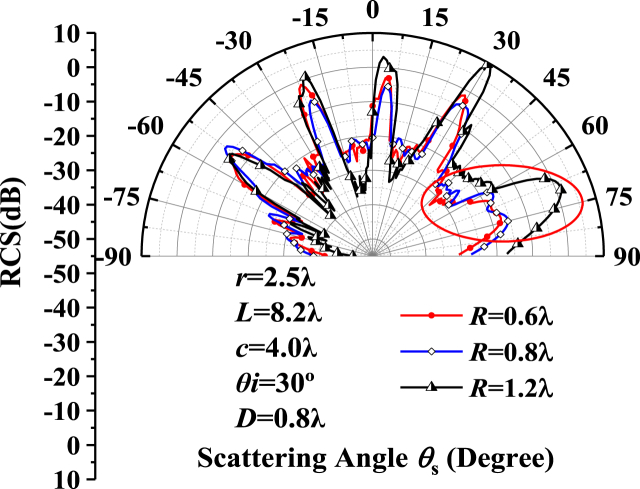
Bistatic radar cross section angle distribution on the single-crystal silicon ellipsoid metasurface with different size of pore defects.

**Fig. 9:**
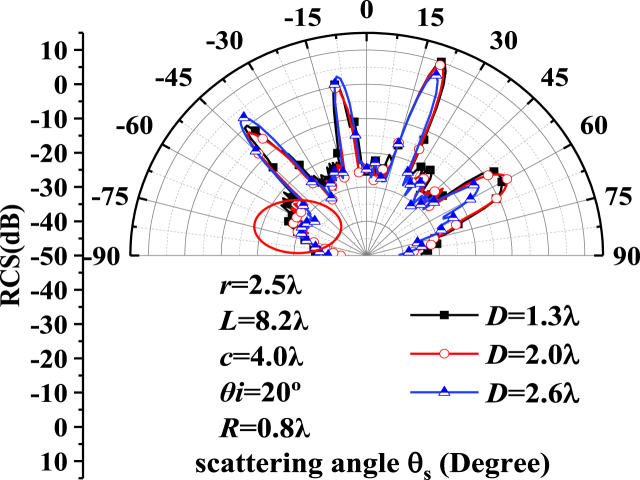
Bistatic radar cross section angle distribution on the single-crystal silicon ellipsoid metasurface with different depth of pore defects.

In the composite scattering model, the parameters directly related to the defects are: defect radius r, burial depth D. The parameters in sections 3.4, 3.5 are as follows: the incident wavelength λ is 0.633 μm; the single-crystal silicon rotary ellipsoidal metasurfaces is 5*5 array structure, the refractive index of the substrate single-crystal silicon is 3.88 + 0.02i, and the unit size is 2.5λ*2.5λ*4.0λμm. The defect is a vacuum pore, and its refractive index is 1.0. Fig. 6 shows illustration of superstructure surface unit structure and defect characteristic parameters. The detailed influences of structural characteristic parameters on the scattering field of the superstructure surface in Fig. 7, Fig. 8, Fig. 9 are shown.

structure and defect characteristic parameters section angle distribution on the single-crystal silicon with different axis ratios ellipsoidal units Metasurface.

The angular distribution of the bistatic radar cross section angle distribution on the single crystal silicon with different axis ratios ellipsoidal units metasurfaces is shown in Fig. 7. The angle of incidence is $\theta_{i}=0^{\circ }$ and the radius of the ellipsoid *r* = 2.5λ. The distance between the centers of adjacent units *L* = 8.2λμm, *c/r* = 0.1,0.5,1.2,1.5; the radius of pore defect *R* = 0.1λ. The green line in the figure represents the calculation result of the micro-rough optical surface, a correlation length *l* and root mean square height δ are 0.43 μm and 0.051 μm, respectively.

It can be seen from Fig. 7 that the decreased RCS between 30° and 60° is attributed to the energy loss caused by the interaction of the pore defect with the nearby rotating ellipsoidal particle unit structure. When *c/r* > 1.0, unit is an oblate rotating ellipsoid, and the peak caused by the periodic unit is very obvious. When *c/r* < 1.0, unit is a flat ellipsoid, and the peak is smooth. Since the radius of the slew ellipsoid is fixed, the smaller *c/r* ratio is, the smaller the size of the unit becomes. When the ratio of the long and short axes of the ellipsoidal unit on the metasurfaces is small enough, the change trend almost coincidents with that of the radar scattering cross section of the micro-rough optical substrate, such as *c/r* = 0.1. Throughout the results of several parameters, it can be obtained that the results are most typical when *c/r* is close to 1.0. In engineering applications, the intensity of the light field can be adjusted by adjusting the size and height of the unit. The above conclusions provide theoretical reference and basis for the design of periodic structure surfaces in engineering.

### 3.5 The influence of defects sizes and depths on the scattering field of superstructure surface

Fig. 8 gives the results of the Bistatic radar cross section angle distribution on the single-crystal silicon ellipsoid metasurface with different size of vacuum defect particles. The radius of the ellipsoid *r* = 2.5λ, and the distance between the centers of adjacent units *L* = 8.2λ, the long axis *c* = 4.0λ, and the incident angle $\theta_{i}=30^{\circ }$. The vacuum defect particle burial depth is *D* = 0.8λ = 0.5 μm, and the defect size is *R* = 0.6λ,0.8λ,1.2λ, respectively.

Fig. 8 indicates that the scattering field trend increases as the radius of the vacuum defect particle increases. When the vacuum defect radius *R* is comparable to the burial depth *D* (i.e., the blue line), the result in the scattering angle range from 30° to 90° is the scattering interference area of pore defects. The five peaks come from the five periodic structure units. With the pore defect radius *R* is greater than the burial depth *D*, double peak gradually degraded to single peak. The detection of light field surface scattering field overall increase while the peak is very obvious (as shown in the red circle range), because the state of defect is from the buried into inlaid. The largest difference in the angular distribution of the radar scattering cross-section occurs at 65°, where the main contribution to the difference field comes from the defect state, which is from the body scattering to surface scattering. The contribution field of defect position is dominant from physical representation. In practical engineering, the situation of defect particles can be preliminarily determined based on the RCS of composite scattering field.

Fig. 9 illustrates the bistatic radar cross section angle distribution on the single-crystal silicon ellipsoid metasurface with different depth of pore defects. The incident angle is $20^{\circ }$, the radius of the ellipsoid is 2.5λ, the distance between the centers of adjacent units is 8.2λ, the long axis *c* is 4.0λ, and the defect particle size *R* is 0.8λ, and the burial depths *D* are1.3λ,2.0λ,2.6λ, respectively.

As can be seen from Fig. 9, the peak point corresponds to a conversion of 20° because the incident angle is adjusted to 20°. In the range of detection angle from 0° to 90°, the deeper the defect is buried, the smaller the total field strength becomes. Because when the burial depth D is greater, there is a longer interacting with the metasurface, resulting in a higher consumption of light energy. When the burial depth *D* is less than 2.0λ, the maximum occurs in the incident direction. The extreme value is similar, the burial depth has little impact on the composite scattering field. The peak at the closest part of the defect disappears and the light field energy is lost due to the interaction between the pore defect and its nearest unit (i.e., the red circle region in the figure). In engineering, the defect state is further diagnosed accurately based on the peak position. The defects can be used to enhance the local light field, focus and adjust the direction of light field and other meaningful work such as designing advanced optical devices or enhancing sensing capabilities.

## 4 Conclusions

In this paper, we use the MRTD method to investigate the scattering problem of ellipsoidal metasurfaces with pore defects. The presence of defects disrupts the symmetry of the metasurface. The structural features and defects of the unit significantly impact the properties of the metasurface. Therefore, it is of great significance to research the physical and structural parameters of the unit structure as well as the defect characteristics' parameters on the scattering field of single crystal silicon metasurface. Based on the Maxwell equation, the concept of multiresolution is introduced to establish composite scattering model between the micro-nano-periodic superstructure and the defects, and derive a composite scattering field for comparison with the CST and FDTD method. The influence of defect size and burial depth on the composite light scattering characteristics is analyzed through numerical calculation, and the conclusions are drawn as follows: 1) The scattering contribution of interface roughness tends to independently contribute to the overall field. And the correlation length and root mean square height of a rough surface are crucial parameters which determine the overall performance. 2) The influence of the unit structure: the peak of the light field is smoothed out as *c/r* becomes smaller, and when *c/r* is close to 0.1, the effect of the periodic superstructure on the light field can be ignored. 3) The effect of defect size and burial depth: as the defect size increases and transitions from being buried to being inlaid, the field strength increases significantly, with a very noticeable peak. The presence of defects affects the location and the peak of bistatic radar cross section angle distribution, and the light field energy is lost due to the presence of pore defects and their interaction with the ellipsoidal unit. The above laws and conclusions are applied to engineering problems in order to precisely regulate the periodic superstructure surface, strictly control the quality, and then adjust the optical properties of the system in spectral and spatial dimensions, providing theoretical support and technical support for the realization of ultra-sensitive detection, functional surface design, and scattering peak direction selection.

Aiming at the hot topics of super-surface optical field regulation in recent years, the influence of defects on the metasurfaces optical system with simple structure is studied by numerical method in this paper. The complex structure, spaces, and other multi-dimensions, as well as the effect of roughness on the composite optical field in the metasurface coated with a micro-rough film system, will be further researched in conjunction with an experimental series of work. As a defective three-dimensional metamaterial, it offers potential for flexible control over parameters such as amplitude, phase, and polarization state across multiple bands of waves (e.g. laser, infrared light, terahertz waves, ect.) in future research directions. This presents a novel platform for the miniaturization and high-performance design of optical devices.

## CRediT authorship contribution statement

**Zhi-qiang Yang:** Writing – original draft, Conceptualization. **Juan Chen:** Writing – original draft, Data curation. **Li-guo Wang:** Writing – original draft, Formal analysis. **Li-hong Yang:** Supervision, Methodology. **Yao Li:** Software, Conceptualization. **Zhen-sen Wu:** Visualization, Conceptualization. **Lei Gong:** Writing – review & editing.

## Declaration of competing interest

The authors declare that they have no known competing financial interests or personal relationships that could have appeared to influence the work reported in this paper.
